# Molecular Connectivity Predefines Polypharmacology: Aliphatic Rings, Chirality, and sp^3^ Centers Enhance Target Selectivity

**DOI:** 10.3389/fphar.2017.00552

**Published:** 2017-08-28

**Authors:** Stefania Monteleone, Julian E. Fuchs, Klaus R. Liedl

**Affiliations:** Institute of General, Inorganic and Theoretical Chemistry, Center of Molecular Biosciences, University of Innsbruck Innsbruck, Austria

**Keywords:** dark chemical matter, drug discovery, molecular descriptors, stereochemistry, chemical properties, screening library design, off-targets, drug repurposing

## Abstract

Dark chemical matter compounds are small molecules that have been recently identified as highly potent and selective hits. For this reason, they constitute a promising class of possible candidates in the process of drug discovery and raise the interest of the scientific community. To this purpose, Wassermann et al. (2015) have described the application of 2D descriptors to characterize dark chemical matter. However, their definition was based on the number of reported positive assays rather than the number of known targets. As there might be multiple assays for one single target, the number of assays does not fully describe target selectivity. Here, we propose an alternative classification of active molecules that is based on the number of known targets. We cluster molecules in four classes: black, gray, and white compounds are active on one, two to four, and more than four targets respectively, whilst inactive compounds are found to be inactive in the considered assays. In this study, black and inactive compounds are found to have not only higher solubility, but also a higher number of chiral centers, sp^3^ carbon atoms and aliphatic rings. On the contrary, white compounds contain a higher number of double bonds and fused aromatic rings. Therefore, the design of a screening compound library should consider these molecular properties in order to achieve target selectivity or polypharmacology. Furthermore, analysis of four main target classes (GPCRs, kinases, proteases, and ion channels) shows that GPCR ligands are more selective than the other classes, as the number of black compounds is higher in this target superfamily. On the other side, ligands that hit kinases, proteases, and ion channels bind to GPCRs more likely than to other target classes. Consequently, depending on the target protein family, appropriate screening libraries can be designed in order to minimize the likelihood of unwanted side effects early in the drug discovery process. Additionally, synergistic effects may be obtained by library design toward polypharmacology.

## Introduction

Drug discovery for a specific target is a long process that starts from hit finding: in the past high throughput screening (HTS) of huge compound libraries was the most common process in pharmaceutical companies. However, the chemical space that the HTS can reach is restricted to the molecules that were previously synthesized and included in the screened library. This certainly precludes the discovery of new compounds, as the chemical space is much wider and the use of limited knowledge makes the hit discovery challenging (Dobson, 2004; Reymond, 2015).

To overcome these disadvantages, computational techniques can be applied in order to speed up the process of drug design and to perform *de novo* drug design. One of the most popular methods is virtual screening, that is the identification of possible candidates for assays by considering their molecular properties (ligand-based) and/or their interactions with the macromolecular binding partner (typically a protein) when its structure is available (structure-based) (Kirchmair et al., 2009; von Grafenstein et al., 2014; Kaserer et al., 2015; Vuorinen and Schuster, 2015). Different virtual compound libraries can be designed, depending on the target properties and on the desired pharmacokinetics (Lionta et al., 2014). Therefore, fragment-based and relatively small focused libraries have found great success: a wider chemical space is covered by virtually assembling many different building blocks as in combinatorial synthesis (Chevillard and Kolb, 2015; Reymond, 2015) or by building compounds directly starting from the structure complex with the first fragment (Srinivas Reddy et al., 2013).

Furthermore, virtual libraries can be properly designed in order to identify active compounds, which also exhibit suitable ADMET (absorption, distribution, metabolism, excretion, and toxicity) properties (Gleeson, 2008). The Lipinski’s rule of five (Lipinski, 2004) helps in identifying orally active compounds, but does not fully describe all facets of druggability. For instance, today the *in silico* assessment of molecular toxicity is still challenging (Roncaglioni et al., 2013; Raies and Bajic, 2016), but at the same time necessary to establish early and *in silico* if a molecule could cause toxic side effects, rather than in the later preclinical phase by experimental assays, which are expensive and time consuming (Peters et al., 2012). On one side, it is undoubted that side effects take place when a molecule is active on multiple targets and, hence, by definition promiscuous (Wang and Greene, 2012). On the other side, promiscuity can represent also an advantage, where the goal of the drug development is to obtain a polypharmacological effect, especially in the treatment of diseases that involve multiple targets (Anighoro et al., 2014; Rastelli and Pinzi, 2015).

To this purpose, the computation of molecular properties has been established not only to discriminate between inactive and active, weak and potent compounds, but also between promiscuous and selective ligands. For instance, Lovering et al. (2009) showed that target selectivity increases with the number of chiral centers and with higher molecular complexity, described as fraction of carbon sp^3^ atoms. Moreover, the presence of amines and high clogP values negatively affect target selectivity (Lovering, 2013). Indeed, many promiscuous compounds are positively charged at physiological pH, as emerged also from the analysis of a Roche dataset (Peters et al., 2009).

With the recent identification of “dark chemical matter” (DCM) as promising starting point for drug discovery (Macarron, 2015; Wassermann et al., 2015), chemical properties of this potentially highly selective compound species are in the focus of interest. Wassermann et al. (2015) use descriptors based on the two-dimensional (2D) compound structures and describe subtle shifts in their distributions toward higher solubility (logS), lower hydrophobicity (logP), smaller molecular weight (MW) and lower amount of rings for DCM versus compounds that are frequently active in HTS assays (Wassermann et al., 2015). They define DCM as molecules that are inactive in at least 100 assays, presuming that these compounds would hit only few possible targets. However, there are compounds, which are listed as DCM, but they are active on many different targets. For example, CID1048281 (Supplementary Figure 1) is considered DCM because it is inactive in more than 650 assays, but it is also active in other six assays in PubChem, which test the activity on unrelated targets (RAR-related orphan receptor gamma, aldehyde dehydrogenase, tyrosyl-DNA phosphodiesterase, ATPase, bromodomain adjacent to zinc finger domain and shiga toxin).

On the other side, many assays may be available for the same target and the number of negative test outcomes does not necessarily correctly depict target selectivity. For example, there are 245 small-molecule bioassays reported on PubChem for the adrenoreceptor beta 1 and more than 350 for the beta 2 subtype. Moreover, most of these bioassays are not specific for a receptor subtype or are simply confirmatory. In order to overcome this pitfall, Wassermann et al. (2015) filtered the set of bioassays by removing redundant readouts for the same target.

As shown, it is extremely hard to determine the target selectivity of a molecule solely on the base of its assay positive or negative outcomes. For this reason, we propose an alternative classification of active molecules, on the base of the number of targets they hit, in order to investigate target selectivity and/or polypharmacology in the early phase of the drug discovery process. In detail, we distinguish between molecules that are selective toward one single protein and other compounds that are active on multiple targets. In this way, it is possible to identify which molecular properties enhance target selectivity and which protein families are likely to constitute off-targets.

## Materials and Methods

### Ligand Dataset Retrieval

We extracted the set of 139,352 DCM compounds from Novartis and PubChem (Kim et al., 2015) as InChi (IUPAC International Chemical Identifier) from the Supporting Information of Wassermann et al. (2015) and downloaded the 3D coordinates of 139,328 molecules from the PubChem Compound database (Kim et al., 2016).

The set of active compounds was extracted from PubChem BioAssay (Wang et al., 2017) using the list of 459 bioassays provided by Wassermann et al. (2015). Active compounds (256,448) were extracted via their compound identifiers (CIDs), downloaded as 3D coordinates (237,510) and pooled to a single set of 376,838 compounds.

Furthermore, we performed a filtering step to remove duplicates within the dataset. To this purpose we used the RDKit (RDKit, 2015) chemoinformatics toolkit. Moreover, we removed the compounds that were active but without any specified targets (14,464). Our final dataset included 341,599 molecules.

### Computation of Molecular Descriptors

The PubChem coordinate files contained already precomputed 2D descriptors, including MW, number of heavy atoms, defined and undefined stereocenters, H-bond donors and acceptors, which were considered for our analysis as provided.

Additionally, we calculated logS (Hou et al., 2004) and logP_(o/w)_ using the MOE (Molecular Operating Environment, version 2015.1001) (MOE, 2016) molecular descriptor tools and the atomic geometries with MOE’s Scientific Vector Language (SVL) function “aGeometry” together with the SMARTS matching function “sm_MatchAll.” In detail, aGeometry returns the hybridization of an atom and sm_MatchAll searches for specific SMARTS patterns, which we used to count non-ring and non-terminal carbon atoms. For instance, sp^3^ carbon atoms are counted by matching “CH_2_” SMARTS codes. In order to restrict the count to non-ring and non-terminal atoms, we specified “!r” and “!H_3_” respectively.

Furthermore, we used RDKit (RDKit, 2015) to count the number of single and fused aromatic and aliphatic rings as well as the number of carbon–carbon and carbon–nitrogen double bonds based on SMILES codes.

Statistical analysis, including the two-sided Wilcoxon rank-sum test and Kolmogorov–Smirnov test, was performed using R (R Development Core Team, 2010) (Supplementary Tables 2–4).

### Target Retrieval and Analysis

Assay and target information for all compounds have been retrieved from the PubChem database by querying the compounds identifiers (CID) against the assay summary webpage. Active targets with specified gene id were considered for Uniprot (Bateman et al., 2015; The UniProt Consortium, 2017) retrieval, in order to convert the gene id to the associated protein‘s Uniprot accession number.

We assigned the protein superfamily for every target, by searching Uniprot accession numbers into lists of GPCRs, kinases, proteases, and ion channels. We obtained the lists of 3,092 GPCRs, 1,365 kinases and 11,606 proteases from Uniprot, and the list of 899 ion channels from ChEMBL (Bento et al., 2014) and IUPHAR/BPS Guide to Pharmacology (Southan et al., 2016).

We counted the number of targets on which a molecule is found to be active and clustered active ligands in three classes: black compounds are active only on one single target, gray compounds are active on two to four targets and white compounds are active on more than four targets. We defined these cut-off values in order to obtain a comparable number of molecules in every subset: 73,383 black, 103,025 gray, 87,303 white, 77,888 inactive compounds (compound set provided via SI).

Figures are generated by using MATLAB (MATLAB, 2012), R (R Development Core Team, 2010) and ChemDraw (PerkinElmer Informatics, 1998–2015).

## Results

### Molecular Descriptors

**FIGURE 1:**
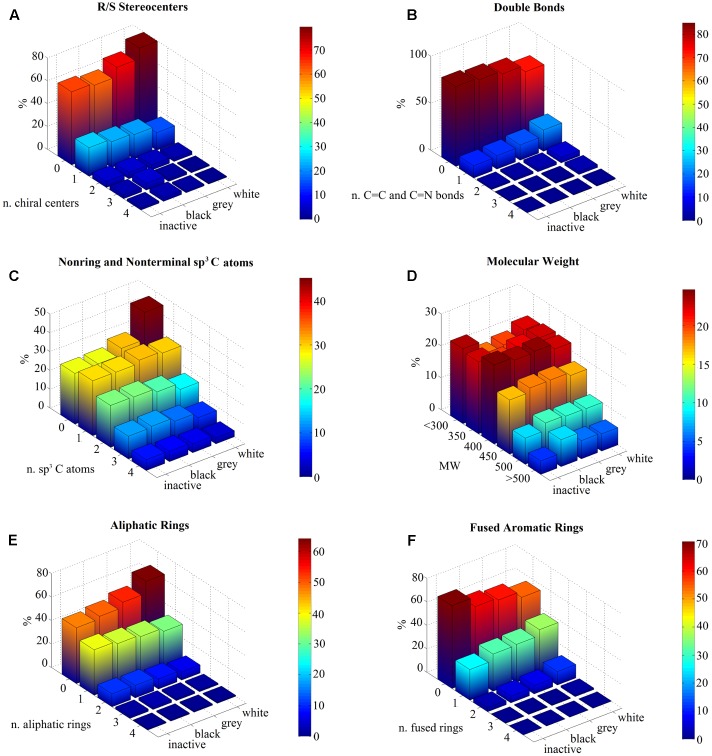
Statistical analysis of molecular descriptors per ligand class (inactive, black, gray, and white). Data are represented as 3D bar plots, colored according to the percentage values for each subset (see color bar). **(A)** The number of R/S stereocenters per molecule shows that most of white compounds have no chiral centers, whereas inactive molecules show the highest percentage of compounds with one stereocenter. **(B)** The number of carbon–carbon or carbon–nitrogen double bonds is higher for white ligands compared to the other classes, which normally have none. **(C)** Inactive and black sets exhibit higher content of non-ring and non-terminal sp^3^ carbon atoms with respect to white compounds, which tend to be sp^2^ hybridized. **(D)** The molecular weight (MW) is similar for all subsets in the range 300–500 Da, but shows different results for smaller and higher values. Indeed, inactive and white compounds exhibit higher percentages for values lower than 300 Da, with respect to black and gray sets. On the contrary, black compounds can be rather complex structures as their MW can be higher than 500 Da. The MW axis is divided into different ranges and its labels represent the highest boundary. For instance, “350” indicates compounds with MW values between 300 and 350. **(E)** Most of white molecules have no aliphatic rings, which characterize instead inactive and black datasets. **(F)** In contrast, a higher number of fused aromatic rings is a chemical feature of white molecules.

We analyzed the distributions of 2D molecular descriptors within the compound sets (inactive, black, gray, and white). We find that chirality enhances target selectivity. For instance, molecules become more selective if they present at least one chiral center: inactive and black compounds contain a higher number of defined R/S stereocenters with respect to white molecules (**Figure 1A**). On the contrary, the absence of a chiral center enhances promiscuity, as described by the percentage of white molecules (∼79% versus ∼62% in black ones) (Supplementary Table 1).

On the opposite, if at least a carbon–carbon or carbon–nitrogen double bond is present, molecules tend to be white and, hence, more promiscuous (**Figure 1B**). Otherwise, if they do not have any double bonds, they tend to be inactive or black (∼85% versus ∼69% in white ones) (Supplementary Table 1).

These findings are also confirmed by the analysis of atomic geometries: non-ring and non-terminal sp^3^ carbon atoms enhance selectivity (**Figure 1C**); about 42% of white compounds do not include any sp^3^ carbon atoms, with respect to ∼27% of inactive and black ones (Supplementary Table 1).

We also computed the molecular descriptors that were reported by Wassermann et al. (2015). However, our results show that the MW is not able to properly describe target selectivity: indeed, black compounds do not follow the expected trend, as they show MWs which are comparable to those of white molecules (**Figure 1D**). This finding disagrees with Wassermann et al. (2015), because our dataset does not include all molecules that were considered in the Novartis analysis, but only those that were reported in the publication. As this descriptor appears dataset dependent, we discarded it.

Additionally, the number of rings differs between these classes: black compounds exhibit higher numbers of aliphatic rings (∼36% of black molecules have one aliphatic ring, with respect to 30% of white ones) (**Figure 1E**). By constrast, white compounds show higher numbers of fused aromatic rings (∼35% with respect to 26% of inactive molecules) (**Figure 1F**). Indeed, more than half of the selective molecules has at least one aliphatic ring (∼53% of inactive and ∼51% of black compounds) and no fused aromatic rings (∼71 of inactive and 62% of black compounds).

**FIGURE 2:**
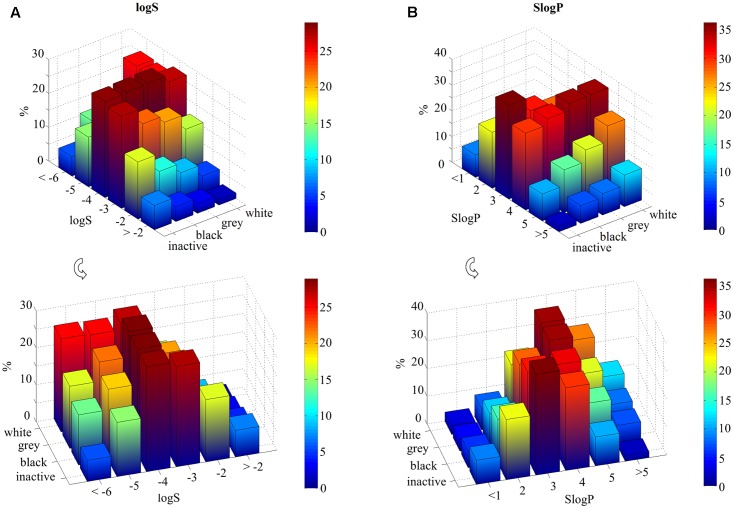
Statistical analysis of molecular solubility (logS) and hydrophobicity (SlogP) per ligand class (inactive, black, gray, and white). Data are represented as 3D bar plots, colored according to the percentage values for each subset (see color bar). The logS and SlogP axes are divided into different ranges and labels represent the highest boundary of each range. **(A)** Molecular solubility, reported as logS, is higher for inactive and black compounds for values higher than *–*4. Whereas white compounds have logS values lower than *–*4. **(B)** White compounds show SlogP values higher than 4. In contrast, inactive and black molecules have values lower than 4.

Furthermore, inactive and black compounds exhibit higher values of logS compared to gray and white compounds, especially for logS in the range between -2 and -4 (**Figure 2A**). By contrast, the opposite trend is observed for lower solubility: half of white molecules shows a logS value lower than -5, whereas only 20% of inactive and ∼30% of black compounds have similar solubility (Supplementary Table 1).

Consequently, lipophilicity increases with the number of targets: gray and white molecules show higher SlogP values than inactive and black ones (**Figure 2B**). For instance, ∼36% of white compounds show SlogP values that are higher than 4, whereas selective molecules (∼33% of inactive and ∼29% of black compounds) exhibit SlogP values which are in the range between 2 and 3.

Calculating these molecular descriptors, it is possible to predict which building blocks characterize black compounds and, therefore, can be used for synthesis of new selective drug candidates.

### Target Analysis

**FIGURE 3:**
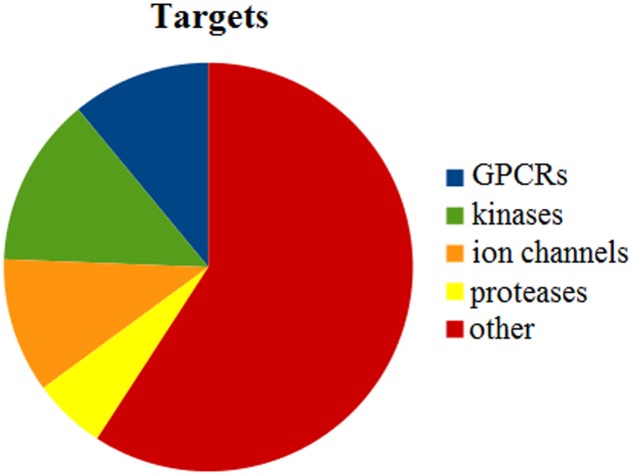
Statistical analysis of targets that are present in the entire dataset. In total, we identified 2,715 different targets. GPCRs represent 10.98%, kinases 13.41%, ion channels 10.68%, and proteases 5.78%. Other targets include further enzymes, nuclear receptors, and transcription factors.

Our dataset includes ligands that bind to a variety of targets, 2,715 in total. For instance, 10.98% of the targets are represented by G-protein coupled receptors (GPCRs), 13.41% by kinases, 10.68% by ion channels and 5.78% by proteases (**Figure 3**). About 60% of the targets comprise other enzymes, receptors or transcription factors that do not fall into these four major target classes.

**FIGURE 4:**
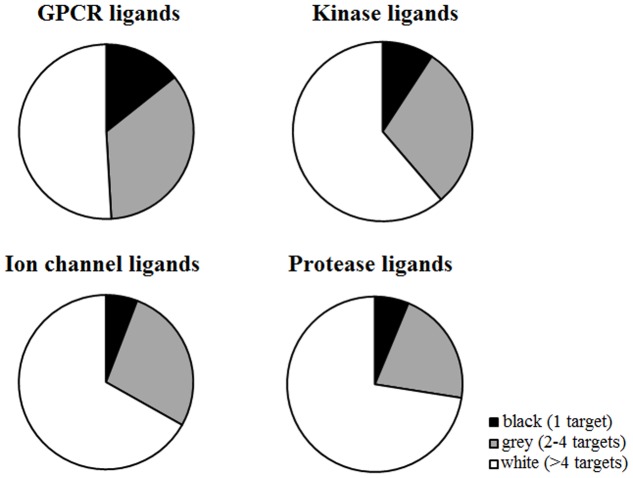
Distribution of black, gray, and white compounds in every target class. The number of black compounds is higher for GPCR ligands (14.30%) compared to other targets (5.80% ion channels, 6.25% ion channels, 9.21% kinases). In contrast, ion channels and proteases have higher percentages of white molecules.

**FIGURE 5:**
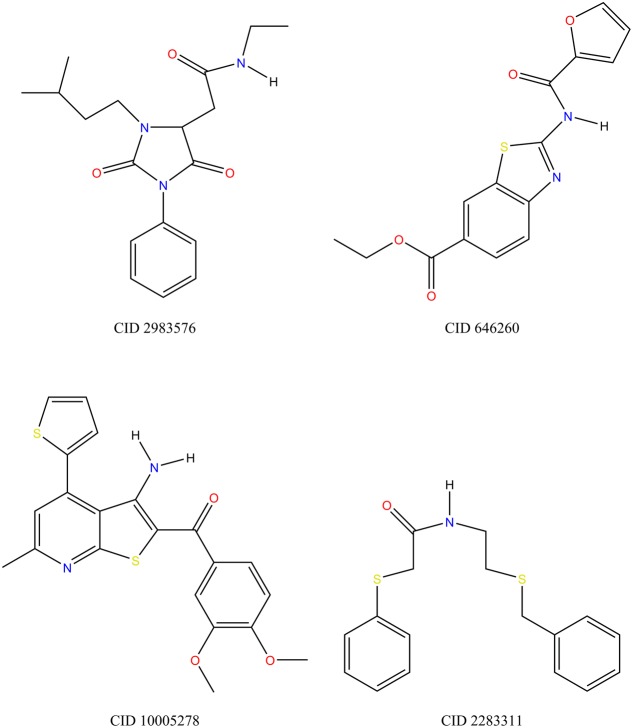
Ligands that represent the dataset. Compounds are labeled according to the compound identifier (CID) from PubChem. CID 2983576 is a selective GPCR ligand: its absolute stereochemistry is undefined in PubChem and, hence, not shown here. CID 646260 is a protease ligand, which binds also to other non-protease targets. CID 1005278 is a kinase ligand that binds also to other non-kinase targets. CID 2283311 is a selective kinase ligand that is active only on one target.

G-protein coupled receptor ligands are more selective than other classes, as the number of black compounds is higher (14.30%) with respect to other targets (5.80% ion channels, 6.25% ion channels, 9.21% kinases) (**Figure 4**). For example, CID 2983576 is a ligand that binds to the human cholinergic muscarinic receptor 4 and is inactive toward other muscarinic receptor subtypes (**Figure 5**). As many other black compounds, it contains a chiral center, an aliphatic ring, several non-ring and non-terminal sp^3^ carbon atoms (5) and has a low logP value (2.2).

**FIGURE 6:**
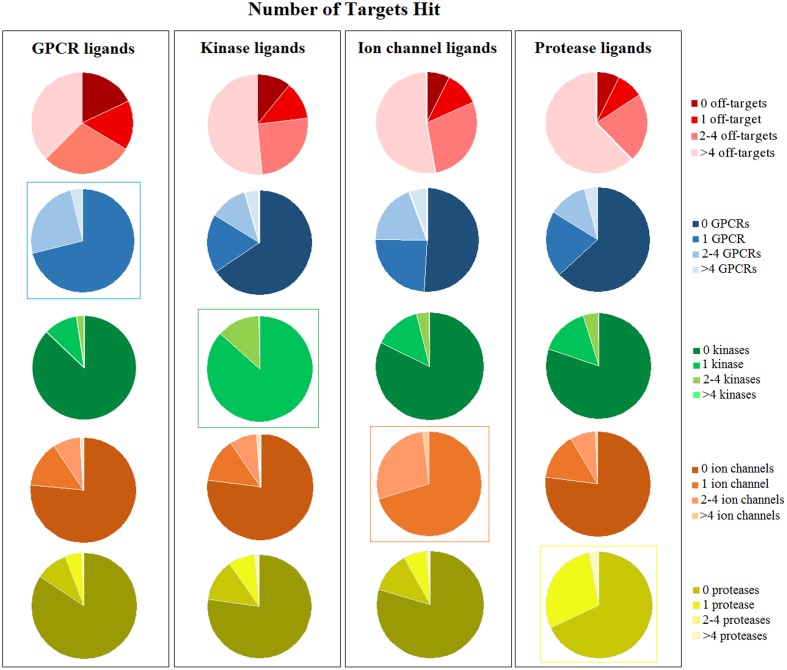
Number of targets hit by every ligand class (GPCRs, kinases, ion channels, and proteases). The first row shows if ligands can hit other targets that are not included in their own target class: for instance, GPCR ligands might hit only GPCRs (indicated as “0 off-targets”) or also other non-GPCR targets (larger number of off-targets). The following rows of pie charts show the number of ligands that hit a specific target class: for instance, GPCR ligands hit at least one GPCR, whereas kinase or ion channel or protease ligands can hit GPCRs or not (indicated as “0 GPCRs”). The same is shown for all four target classes. Intra-class selectivity is highlighted by colored boxes around the pie charts.

Ligands that bind to ion channels and proteases tend to be more promiscuous (**Figure 4**). This is particularly pronounced for proteases, where 62% of ligands can bind to more than four non-protease targets (**Figure 6**). For example, CID 646260 is active on caspase 3 and other non-protease targets, such as GPCRs and other enzymes.

In contrast, only 37% of GPCR ligands binds to other proteins beyond GPCRs. For instance, only 13% of GPCR ligands bind to kinases, 16% to proteases and 24% to ion channels.

Instead, kinase ligands are able to bind to many non-kinase targets. For example, compound CID 1005278 binds not only to kinases (such as RIPK), but also to potassium channels (such as KCNQ1), dopamine receptors (D1 and D3), proteases and other non-kinase targets. However, analysis of intra-class activity shows that kinase ligands in general bind only to one kinase (for example, CID 2283311 is a black molecule that is active only on MAP3K3). This evidence is surprising, as kinases are known to be promiscuous, especially toward other kinases (Davis et al., 2011). However, the number of kinase ligands in our dataset is relatively small (27,935) and we might miss information from unselective ligands that were not included in the analysis.

Furthermore, ion channel, protease and kinase ligands exhibit higher chances to bind to GPCRs: almost half of ion channel (49%), 36.6% of protease and 35% of kinase ligands bind to GPCRs as well. However, this trend cannot be observed for proteases, kinases or ion channels, as they exhibit lower probabilities to bind to these target classes (Supplementary Figure 2).

## Discussion

The escape from flatland has already been described as a valuable approach to improve clinical success (Lovering et al., 2009) and the unique activity profiles of highly potent and selective molecules might be the underlying principle. It is chemically intuitive that more complex molecular shapes restrict the diversity of binding partners and provide selectivity gains (Mendez-Lucio and Medina-Franco, 2017). A criterion favoring complex 3D shapes, with chiral centers and high sp^3^ carbon contents, low number of double bonds and fused aromatic rings, in candidate molecules might complement widely accepted criteria for drug-likeness solely based on 2D molecular properties, like solubility and MW (Lipinski, 2004; Leeson and Springthorpe, 2007).

We also believe that these molecular properties highly affect the target selectivity. Indeed, already Lovering et al. (2009) stated that the degree of saturation is able to distinguish marketed drugs from drug-like molecules. In detail, compounds that have success through clinical trials are characterized by increased saturation and the presence of chiral centers. For instance, our findings confirm that the sp^3^ conformation is a key feature to obtain target selectivity and in turn to improve clinical success in the process of drug development.

These molecular descriptors, together with solubility and lipophilicity, may be readily applied as an additional selection criterion for promising starting points in early stage drug discovery. Wassermann et al. (2015) have shown DCM is more soluble than active molecules. Our results are in agreement with their findings, as selective compounds are more soluble than promiscuous ones.

In contrast, MW does not properly distinguish between inactive and white molecules as shown in other datasets. For instance, promiscuity is enhanced by lower values of MW in a dataset from Pfizer (Hopkins et al., 2006), but higher values in datasets from Novartis (Azzaoui et al., 2007), Roche (Peters et al., 2009) and Boehringer Ingelheim (Muegge and Mukherjee, 2016).

We also considered further molecular descriptors, such as the number of hydrogen bond donors and acceptors, but they do not allow to distinguish between selective and promiscuous compounds (Supplementary Table 1), as also shown by Novartis (Azzaoui et al., 2007) and Roche (Peters et al., 2009).

In our dataset many ligands are promiscuous and, hence, can effectively hit off-targets, which are represented by all other targets that a molecule can bind besides the intended target (Rudmann, 2013).

However, in our dataset GPCR ligands are highly selective. This evidence appears to be in contrast to previous knowledge, as GPCRs are known to be promiscuous targets, especially if their ligands are not peptidic or small molecules (Paolini et al., 2006). For instance, our results may change by considering specialized datasets, such as PDSP Ki database (Roth et al., 2000).

Additionally, our analysis shows that ligands from other target protein families can easily bind GPCRs. Indeed, there are great overlaps between all four target classes that we considered (Supplementary Figure 2) and we do not know if these molecules were developed firstly as GPCR ligands or not.

The identification of a GPCR as off-target is extremely important, as the activity on specific GPCRs is also related to severe side effects, e.g., cardiovascular diseases. Indeed, 5-HT2B has been identified as cause of valvulopathy and led to the withdrawal of drugs from the market (Huang et al., 2009).

Our results show that protease ligands can bind to many off-targets: indeed, it can be difficult to achieve target selectivity within related proteases (Drag and Salvesen, 2010) but strategies to rationally improve the selectivity profiles of protease inhibitors based on substrate peptide data and experimental 3D structures have been described (Fuchs et al., 2013).

In our dataset, kinase ligands seem to be selective toward only one kinase member rather than to more targets in the same protein family. However, this unexpected outcome can be explained by the relatively low amount of kinases ligands that is present in the dataset. Kinase ligands are indeed generally known to be promiscuous, but some of them exhibit higher selectivity, especially if they bind to the pocket close to the ATP site and prefer a specific conformation of the activation loop (Davis et al., 2011). Moreover, in our dataset we identify even more pronounced polypharmacology within and between other target classes. For instance, ion channel ligands overlap with GPCR ligands, as they frequently exhibit a common ligand scaffold, which includes an amine linked to an aromatic ring by an alkylic chain that is present in benzodiazepines or dihydropyridines. In addition, ion channels constitute a common off-target, causing cardiac adverse effects. Indeed, hERG potassium channels are responsible of arrhythmias, in particular torsades de pointes, and many antipsychotics and other drugs bind to these channels as off-targets, increasing the risk of cardiovascular diseases (Silvestre and Prous, 2007). As example, the antihistaminic terfenadine was withdrawn from the market for its toxic adverse effect, that was caused by this off-target activity (Monahan et al., 1990).

This analysis bring us to ask if we can identify likely off-targets in the early discovery process. Normally, in the early steps, target selectivity is considered only among related targets, which are proteins that belong to the same protein family, since high structure and ligand similarity is expected. In this case, target selectivity can be rationalized, e.g., via X-ray structures of targets and off-targets. However, several adverse side effects are caused by distant or nearly unrelated targets. For this reason, the prediction of ligand binding is still challenging and the use of cheminformatics tools can guide the medicinal chemists in identifying the chemical features that typically cause promiscuity (Besnard et al., 2012). Nevertheless, the training of virtual screening models is limited by the use of biased ligand sets. Indeed, our analysis show that results highly depend on the selected dataset, which affected the distribution of the physico-chemical properties and target classes. Therefore we expect that based on the desired target, specialized datasets can be used to further improve the performance of *in silico* models.

In particular, screening libraries can be properly designed by taking into account molecular properties, such as stereochemistry, atomic geometries and rings, besides solubility and lipophilicity. Many predesigned compound libraries are already freely available online and could be easily filtered or prioritized by using these 2D descriptors, without the need of applying a time consuming and computationally demanding generation of 3D conformers.

## Conclusion

A good starting point for the design of a selective drug should favor aliphatic over aromatic rings, alkylic chains containing sp^3^ carbon atoms over double bonds, and stereocenters over achiral atoms. Even though the introduction of chiral centers can make the synthesis more challenging, the gain in target selectivity may be considerable.

On the other hand, polypharmacology could be achieved by introducing flat chemical moieties, such as fused aromatic rings and double bonds. However, this could bring not only additional desired, but also undesired side effects.

## Author Contributions

SM and JF performed the research. SM, JF, and KL designed the study and contributed to the preparation of the manuscript.

## Conflict of Interest Statement

JF is a permanent employee of Boehringer Ingelheim RCV GmbH & Co KG. The other authors declare that the research was conducted in the absence of any commercial or financial relationships that could be construed as a potential conflict of interest.

## References

[B1] Anighoro A. Bajorath J. Rastelli G. (2014). Polypharmacology: challenges and opportunities in drug discovery. *J. Med. Chem.* 57 7874–7887. 10.1021/jm5006463 24946140

[B2] Azzaoui K. Hamon J. Faller B. Whitebread S. Jacoby E. Bender A. (2007). Modeling promiscuity based on in vitro safety pharmacology profiling data. *Chemmedchem* 2 874–880. 10.1002/cmdc.200700036 17492703

[B3] Bateman A. Martin M. J. O’donovan C. Magrane M. Apweiler R. Alpi E. (2015). UniProt: a hub for protein information. *Nucleic Acids Res.* 43 D204–D212. 10.1093/nar/gku989 25348405 PMC4384041

[B4] Bento A. P. Gaulton A. Hersey A. Bellis L. J. Chambers J. Davies M. (2014). The ChEMBL bioactivity database: an update. *Nucleic Acids Res.* 42 D1083–D1090. 10.1093/nar/gkt1031 24214965 PMC3965067

[B5] Besnard J. Ruda G. F. Setola V. Abecassis K. Rodriguiz R. M. Huang X. P. (2012). Automated design of ligands to polypharmacological profiles. *Nature* 492 215–220. 10.1038/nature11691 23235874 PMC3653568

[B6] Chevillard F. Kolb P. (2015). SCUBIDOO: a large yet screenable and easily searchable database of computationally created chemical compounds optimized toward high likelihood of synthetic tractability. *J. Chem. Inform. Model.* 55 1824–1835. 10.1021/acs.jcim.5b00203 26282054

[B7] Davis M. I. Hunt J. P. Herrgard S. Ciceri P. Wodicka L. M. Pallares G. (2011). Comprehensive analysis of kinase inhibitor selectivity. *Nat. Biotechnol.* 29 1046–U1124. 10.1038/nbt.1990 22037378

[B8] Dobson C. M. (2004). Chemical space and biology. *Nature* 432 824–828. 10.1038/nature03192 15602547

[B9] Drag M. Salvesen G. S. (2010). Emerging principles in protease-based drug discovery. *Nat. Rev. Drug Discov.* 9 690–701. 10.1038/nrd3053 20811381 PMC2974563

[B10] Fuchs J. E. Von Grafenstein S. Huber R. G. Kramer C. Liedl K. R. (2013). Substrate-driven mapping of the degradome by comparison of sequence logos. *PLoS Comput. Biol.* 9:e1003353 10.1371/journal.pcbi.1003353 PMC3828135 24244149

[B11] Gleeson M. P. (2008). Generation of a set of simple, interpretable ADMET rules of thumb. *J. Med. Chem.* 51 817–834. 10.1021/jm701122q 18232648

[B12] Hopkins A. L. Mason J. S. Overington J. P. (2006). Can we rationally design promiscuous drugs? *Curr. Opin. Struct. Biol.* 16 127–136. 10.1016/j.sbi.2006.01.013 16442279

[B13] Hou T. J. Xia K. Zhang W. Xu X. J. (2004). ADME evaluation in drug discovery. 4. Prediction of aqueous solubility based on atom contribution approach. *J. Chem. Inf. Comput. Sci.* 44 266–275. 10.1021/ci034184n 14741036

[B14] Huang X. P. Setola V. Yadav P. N. Allen J. A. Rogan S. C. Hanson B. J. (2009). Parallel functional activity profiling reveals valvulopathogens are potent 5-hydroxytryptamine(2B) receptor agonists: implications for drug safety assessment. *Mol. Pharmacol.* 76 710–722. 10.1124/mol.109.058057 19570945 PMC2769050

[B15] Kaserer T. Beck K. R. Akram M. Odermatt A. Schuster D. (2015). Pharmacophore models and pharmacophore-based virtual screening: concepts and applications exemplified on hydroxysteroid dehydrogenases. *Molecules* 20 22799–22832. 10.3390/molecules201219880 26703541 PMC6332202

[B16] Kim S. Thiessen P. A. Bolton E. E. Bryant S. H. (2015). PUG-SOAP and PUG-REST: web services for programmatic access to chemical information in PubChem. *Nucleic Acids Res.* 43 W605–W611. 10.1093/nar/gkv396 25934803 PMC4489244

[B17] Kim S. Thiessen P. A. Bolton E. E. Chen J. Fu G. Gindulyte A. (2016). PubChem substance and compound databases. *Nucleic Acids Res.* 44 D1202–D1213. 10.1093/nar/gkv951 26400175 PMC4702940

[B18] Kirchmair J. Distinto S. Markt P. Schuster D. Spitzer G. M. Liedl K. R. (2009). How to optimize shape-based virtual screening: choosing the right query and including chemical information. *J. Chem. Inform. Model.* 49 678–692. 10.1021/ci8004226 19434901

[B19] Leeson P. D. Springthorpe B. (2007). The influence of drug-like concepts on decision-making in medicinal chemistry. *Nat. Rev. Drug Discov.* 6 881–890. 10.1038/nrd2445 17971784

[B20] Lionta E. Spyrou G. Vassilatis D. K. Cournia Z. (2014). Structure-based virtual screening for drug discovery: principles, applications and recent advances. *Curr. Top. Med. Chem.* 14 1923–1938. 10.2174/1568026614666140929124445 25262799 PMC4443793

[B21] Lipinski C. A. (2004). Lead- and drug-like compounds: the rule-of-five revolution. *Drug Discov. Today Technol.* 1 337–341. 10.1016/j.ddtec.2004.11.007 24981612

[B22] Lovering F. (2013). Escape from Flatland 2: complexity and promiscuity. *Medchemcomm* 4 515–519. 10.1039/c2md20347b

[B23] Lovering F. Bikker J. Humblet C. (2009). Escape from Flatland: increasing saturation as an approach to improving clinical success. *J. Med. Chem.* 52 6752–6756. 10.1021/jm901241e 19827778

[B24] Macarron R. (2015). Chemical libraries: how dark is HTS dark matter? *Nat. Chem. Biol.* 11 904–905. 10.1038/nchembio.1937 26479440

[B25] MATLAB (2012). *Matlab R2012a*. Natick, MA: The MathWorks Inc.

[B26] Mendez-Lucio O. Medina-Franco J. L. (2017). The many roles of molecular complexity in drug discovery. *Drug Discov. Today* 22 120–126. 10.1016/j.drudis.2016.08.009 27575998

[B27] MOE (2016). *Molecular Operating Environment, 2015.1001.* Montreal, QC: Chemical Computing Group Inc.

[B28] Monahan B. P. Ferguson C. L. Killeavy E. S. Lloyd B. K. Troy J. Cantilena L. R. (1990). Torsades-de-pointes occurring in association with terfenadine use. *JAMA* 264 2788–2790. 10.1001/jama.1990.03450210088038 1977935

[B29] Muegge I. Mukherjee P. (2016). Performance of dark chemical matter in high throughput screening. *J. Med. Chem.* 59 9806–9813. 10.1021/acs.jmedchem.6b01038 27762554

[B30] Paolini G. V. Shapland R. H. B. Van Hoorn W. P. Mason J. S. Hopkins A. L. (2006). Global mapping of pharmacological space. *Nat. Biotechnol.* 24 805–815. 10.1038/nbt1228 16841068

[B31] PerkinElmer Informatics (1998–2015). *ChemDraw 15.0.* Available at: http://media.cambridgesoft.com/support/15/ChemDrawHelp.pdf

[B32] Peters J. U. Hert J. Bissantz C. Hillebrecht A. Gerebtzoff G. Bendels S. (2012). Can we discover pharmacological promiscuity early in the drug discovery process? *Drug Disc. Today* 17 325–335. 10.1016/j.drudis.2012.01.001 22269136

[B33] Peters J. U. Schnider P. Mattei P. Kansy M. (2009). Pharmacological promiscuity: dependence on compound properties and target specificity in a set of recent roche compounds. *Chemmedchem* 4 680–686. 10.1002/cmdc.200800411 19266525

[B34] R Development Core Team (2010). *R: A Language and Environment for Statistical Computing*, 3.2.2 Edn Vienna: R Foundation for Statistical Computing.

[B35] Raies A. B. Bajic V. B. (2016). In silico toxicology: computational methods for the prediction of chemical toxicity. *Wiley Interdiscip. Rev. Comput. Mol. Sci.* 6 147–172. 10.1002/wcms.1240 27066112 PMC4785608

[B36] Rastelli G. Pinzi L. (2015). Computational polypharmacology comes of age. *Front. Pharmacol.* 6:157 10.3389/fphar.2015.00157 PMC4516879 26283966

[B37] RDKit (2015). *RDKit: Open-Source Chemoinformatics, 2015.03.1.* Available at: www.rdkit.org

[B38] Reymond J. L. (2015). The chemical space project. *Acc. Chem. Res.* 48 722–730. 10.1021/ar500432k 25687211

[B39] Roncaglioni A. Toropov A. A. Toropova A. P. Benfenati E. (2013). In silico methods to predict drug toxicity. *Curr. Opin. Pharmacol.* 13 802–806. 10.1016/j.coph.2013.06.001 23797035

[B40] Roth B. L. Lopez E. Patel S. Kroeze W. K. (2000). The multiplicity of serotonin receptors: uselessly diverse molecules or an embarrassment of riches? *Neuroscientist* 6 252–262. 10.1177/107385840000600408

[B41] Rudmann D. G. (2013). On-target and off-target-based toxicologic effects. *Toxicol. Pathol.* 41 310–314. 10.1177/0192623312464311 23085982

[B42] Silvestre J. S. Prous J. R. (2007). Comparative evaluation of hERG potassium channel blockade by antipsychotics. *Methods Find. Exp. Clin. Pharmacol.* 29 457–465. 10.1358/mf.2007.29.7.1119172 17982510

[B43] Southan C. Sharman J. L. Benson H. E. Faccenda E. Pawson A. J. Alexander S. P. H. (2016). Y The IUPHAR/BPS Guide to PHARMACOLOGY in 2016: towards curated quantitative interactions between 1300 protein targets and 6000 ligands. *Nucleic Acids Res.* 44 D1054–D1068. 10.1093/nar/gkv1037 26464438 PMC4702778

[B44] Srinivas Reddy A. Chen L. Zhang S. (2013). “Structure-based de novo drug design,” in *De Novo Molecular Design*, ed. Schneider G. (Weinheim: Wiley-VCH Verlag GmbH & Co. KGaA).

[B45] The UniProt Consortium (2017). UniProt: the universal protein knowledgebase. *Nucleic Acids Res.* 45 D158–D169. 10.1093/nar/gkw1099 27899622 PMC5210571

[B46] von Grafenstein S. Fuchs J. E. Liedl K. R. (2014). “How to profit from molecular dynamics-based ensemble docking,” in *Application of Computational Techniques in Pharmacy and Medicine*, eds Gorb L. Kuz’min V. Muratov E. (Dordrecht: Springer), 501–538.

[B47] Vuorinen A. Schuster D. (2015). Methods for generating and applying pharmacophore models as virtual screening filters and for bioactivity profiling. *Methods* 71 113–134. 10.1016/j.ymeth.2014.10.013 25461773

[B48] Wang X. Y. Greene N. (2012). Comparing measures of promiscuity and exploring their relationship to toxicity. *Mol. Inform.* 31 145–159. 10.1002/minf.201100148 27476959

[B49] Wang Y. Bryant S. H. Cheng T. Wang J. Gindulyte A. Shoemaker B. A. (2017). PubChem BioAssay: 2017 update. *Nucleic Acids Res.* 45 D955–D963. 10.1093/nar/gkw1118 27899599 PMC5210581

[B50] Wassermann A. M. Lounkine E. Hoepfner D. Le Goff G. King F. J. Studer C. (2015). Dark chemical matter as a promising starting point for drug lead discovery. *Nat. Chem. Biol.* 11 958–966. 10.1038/nchembio.1936 26479441

