# Supplementary material for: Molecular Connectivity Predefines Polypharmacology: Aliphatic Rings, Chirality, and sp3 Centers Enhance Target Selectivity
Source: Front Pharmacol. 2017 Aug 28;8:552. doi: 10.3389/fphar.2017.00552 (PMC5581349; doi:10.3389/fphar.2017.00552)
Supplement: Supplementary file 1 [file Table_1.pdf]

Supporting Information

SI Table 1: statistical analysis of molecular descriptors in every ligand class: highlighted values are discussed in Results and Discussion sessions.

Descriptors	N.	Inactive		Black		Grey		White	
		Count	%	Count	%	Count	%	Count	%
Stereocenters	0	49600	63.68	45132	61.50	71735	69.63	68590	78.57
	1	19351	24.84	16343	22.27	21733	21.09	13444	15.40
	2	4164	5.35	4099	5.59	4747	4.61	2639	3.02
	3	2588	3.32	4394	5.99	2343	2.27	1041	1.19
	4	1396	1.79	2668	3.64	1754	1.70	819	0.94
Double bonds	0	66141	84.92	60662	82.66	81725	79.33	60118	68.86
	1	10170	13.06	10492	14.30	16639	16.15	18752	21.48
	2	1234	1.58	1540	2.10	3079	2.99	4699	5.38
	3	268	0.34	477	0.65	1113	1.08	2569	2.94
	4	54	0.07	153	0.21	349	0.34	885	1.01
sp^3 carbon atoms	0	20993	26.95	19856	27.06	31233	30.32	36372	41.66
	1	22643	29.07	21108	28.76	31244	30.33	24257	27.78
	2	17270	22.17	16157	22.02	20678	20.07	13991	16.03
	3	9383	12.05	9301	12.67	11014	10.69	6807	7.80
	4	4456	5.72	4030	5.49	5015	4.87	3061	3.51
Molecular weight	<300	18256	23.44	13706	18.68	20387	19.79	19336	22.15
	300≤x<350	17540	22.52	14963	20.39	22573	21.91	19899	22.79
	350≤x<400	19261	24.73	17456	23.79	24857	24.13	19533	22.37
	400≤x<450	13196	16.94	13391	18.25	18845	18.29	15042	17.23
	450≤x<500	6357	8.16	7687	10.48	10490	10.18	8819	10.10
	≥500	3278	4.21	6180	8.42	5873	5.70	4674	5.35
Aliphatic rings	0	36956	47.45	36431	49.65	55648	54.01	53429	61.20
	1	30046	38.58	26590	36.23	35720	34.67	26290	30.11
	2	8565	11.00	7800	10.63	8755	8.50	5705	6.53
	3	1509	1.94	1594	2.17	1580	1.53	972	1.11
	4	599	0.77	696	0.95	977	0.95	602	0.69
Fused aromatic rings	0	54915	70.51	45573	62.10	61936	60.12	45645	52.28
	1	20255	26.01	23199	31.61	33138	32.17	30957	35.46
	2	2568	3.30	4162	5.67	7270	7.06	9478	10.86
	3	138	0.18	398	0.54	617	0.60	1130	1.29
	4	11	0.01	48	0.07	62	0.06	82	0.09
logS	>-2	5609	7.20	3244	4.42	2949	2.86	1449	1.66
	-3<x≤-2	13122	16.85	8533	11.63	9079	8.81	4700	5.38
	-4<x≤-3	21145	27.15	16530	22.53	21151	20.53	14001	16.04
	-5<x≤-4	22124	28.40	21049	28.68	29805	28.93	23429	26.84
	-6<x≤-5	11431	14.68	14070	19.17	22720	22.05	21715	24.87
	≤-6	4457	5.72	9957	13.57	17321	16.81	22009	25.21
SlogP	<1	6769	8.69	4382	5.97	4333	4.21	2849	3.26
	1≤x<2	16048	20.60	10975	14.96	11602	11.26	6468	7.41
	2≤x<3	26087	33.49	21231	28.93	27012	26.22	17897	20.50
	3≤x<4	20627	26.48	21269	28.98	32676	31.72	28257	32.37
	4≤x<5	6723	8.63	10459	14.25	18865	18.31	21098	24.17
	≥5	1634	2.10	5067	6.90	8537	8.29	10734	12.30

H-bond acceptors	0	17	0.02	18	0.02	26	0.03	24	0.03
	1	956	1.23	843	1.15	1354	1.31	1384	1.59
	2	4543	5.83	3786	5.16	6374	6.19	5789	6.63
	3	10130	13.01	8702	11.86	13377	12.98	12413	14.22
	4	15432	19.81	13841	18.86	20252	19.66	17660	20.23
	5	16309	20.94	15225	20.75	21530	20.90	17838	20.43
	6	13825	17.75	13238	18.04	17779	17.26	14364	16.45
	7	8854	11.37	8690	11.84	11623	11.28	8980	10.29
	8	4662	5.99	4847	6.61	6096	5.92	4852	5.56
	9	2050	2.63	2288	3.12	2691	2.61	2199	2.52
	10	755	0.97	1004	1.37	1101	1.07	890	1.02
	>10	355	0.46	901	1.23	822	0.80	910	1.04
H-bond donors	0	15556	19.97	14790	20.15	22800	22.13	19029	21.80
	1	36903	47.38	33806	46.07	48281	46.86	38490	44.09
	2	20245	25.99	18667	25.44	24765	24.04	21655	24.80
	3	4069	5.22	4817	6.56	5767	5.60	6180	7.08
	4	782	1.00	1018	1.39	1086	1.05	1352	1.55
	5	217	0.28	155	0.21	161	0.16	264	0.30
	>5	116	0.15	130	0.18	165	0.16	333	0.39

SI Figure 1: DCM ligand that is inactive in more than 650 assays, but active in six other unrelated targets. Therefore, in our clustering it constitutes a white (promiscuous) compound.

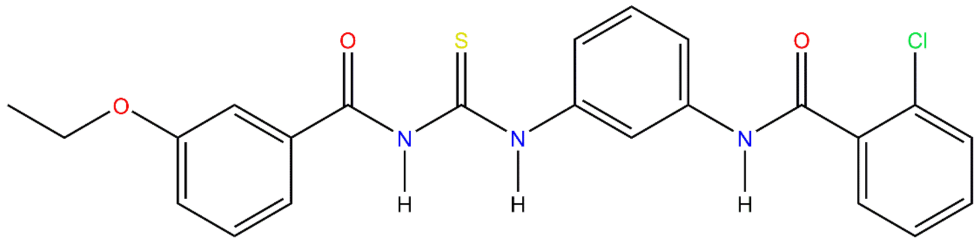

CID 1048281

SI Figure 2: Venn diagram that shows ligand overlaps between the different four target classes.

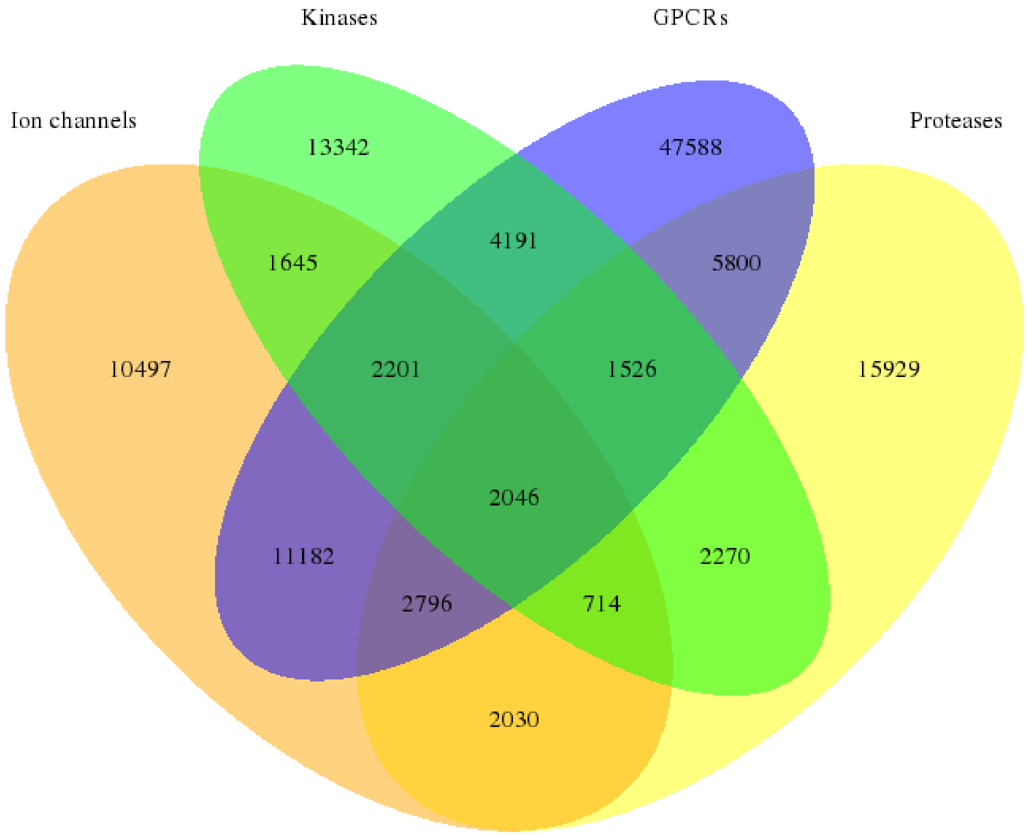

SI Table 2: Differences in molecular properties between the inactive or black compounds and the white set. Two-sided Wilcoxon rank sum tests were performed with R, only for the descriptors with continuous distribution of values. Negative values of the confidence intervals at a confidence level of 0.95 indicate that the median property value is lower for selective molecules and higher for promiscuous ones, and vice versa. Molecular weight (MW) exhibits opposite confidence intervals in the inactive and black datasets.

Property	Inactive-White		Black-White	
	p-value	confidence interval	p-value	confidence interval
logS	< 2.2 e-16	1.08848; 1.11594	< 2.2 e-16	0.62446; 0.65339
SlogP	< 2.2 e-16	-0.97667; -0.95304	< 2.2 e-16	-0.58076; -0.55549
MW	< 2.2 e-16	-6.76795; -5.00797	< 2.2 e-16	10.03908; 11.94900

SI Table 3: p-values obtained with the Kolmogorov-Smirnov test, performed with R. This is a nonparametric goodness-of-fit test, which is suitable for the analysis of discrete data.

Property	Inactive-White	Black-White
Stereocenters	< 2.2 e-16	< 2.2 e-16
sp^3 carbon atoms	< 2.2 e-16	< 2.2 e-16
Double bonds	< 2.2 e-16	< 2.2 e-16
Aliphatic rings	< 2.2 e-16	< 2.2 e-16
Fused aromatic rings	< 2.2 e-16	< 2.2 e-16
H-bond acceptors	< 2.2 e-16	< 2.2 e-16
H-bond donors	< 2.2 e-16	8.94 e-10

SI Table 4: Average and standard deviation of all descriptors.

	Inactive	Black	Grey	White
logS	-3.940±1.349	-4.430±1.488	-4.669±1.471	-5.081±1.515
SlogP	2.600±1.211	3.027±1.374	3.249±1.323	3.553±1.319
MW	358.275±82.854	379.729±96.983	371.379±87.321	367.670±90.162
stereocenters	0.591±1.086	0.732±1.305	0.488±1.020	0.227±0.699
sp3 carbon atoms	1.559±1.484	1.567±1.514	1.447±1.532	1.078±1.313
double bonds	0.177±0.461	0.217±0.545	0.273±0.625	0.469±0.856
aliphatic rings	0.709±0.837	0.699±0.876	0.619±0.835	0.512±0.792
fused aromatic rings	0.332±0.548	0.449±0.635	0.483±0.659	0.615±0.742
H-bond acceptors	5.088±1.880	5.269±2.014	5.098±1.962	5.027±2.068
H-bond donors	1.215±0.905	1.246±0.958	1.180±0.946	1.254±1.024
